# Editorial: Advances in Genomics and Epigenomics of Social Insects

**DOI:** 10.3389/fgene.2016.00199

**Published:** 2016-11-16

**Authors:** Greg J. Hunt, Juergen R. Gadau

**Affiliations:** ^1^Department of Entomology, Purdue UniversityWest Lafayette, IN, USA; ^2^School of Life Sciences, Arizona State UniversityTempe, AZ, USA

**Keywords:** social evolution, phenotypic plasticity, caste determination, epigenetics, eusociality, social insect, reproductive caste, sterile caste

The adaptive advantage of the eusocial lifestyle is evident from the fact that social insects represent more than half of the world's arthropod biomass. This topic explores how the recent advances in genomics and epigenomics are helping researchers to ask and answer questions concerning the evolution of social behavior and the genetic and epigenetic mechanisms behind phenotypic plasticity, i.e., how environmental signals can morph the same genome in a reproductive or non-reproductive individual resulting in dramatically different phenotypes. The articles in this research topic deal broadly with the evolution of reproductive and sterile castes (workers), mechanisms of caste determination, and the role of epigenetic processes for division of labor. The termites were the first group of insects to evolve eusociality and a thorough review describes what is known about the development of subcastes from a mechanistic perspective (nymphs, workers, soldiers) and the genomic contributions of gut symbionts and their hosts in digestion of wood, and the role of symbionts in host fitness (Scharf). Korb et al. compares the genomes of two termites with contrasting social complexities and symbioses. One of the interesting findings was that gene families involved in chemical communication in other social insects are not expanded in termites with more complex social organization. But transposable elements are, suggesting a role for transposition in social evolution but perhaps also pointing toward other mechanisms.

Darwin had a “special difficulty” understanding how sterile worker castes arose in the social insects and the existence of morphological specializations in individuals that did not have progeny. Epigenetic processes could provide mechanisms to encode these specializations within a worker caste just as it does in clonal cells of developing tissues. For example, experimental manipulations that cause honeybee workers to switch task specializations are marked by specific methylation events (Herb). However, the function of gene body methylation in regards to behavioral plasticity of workers, although associated with alternative splicing remains uncertain. The less-studied, and less abundant 5hydroxymethylcytosine (5hmC) modifications are intriguingly enriched in germ cells and brain of honeybees just as they are in mammals (Rasmussen and Amdam). Ruden et al. continue Herb's answer to Darwin's dilemma by suggesting that solitary ancestors of social bees may have experienced nutrient limitations, leading to a de-facto sterile caste in communal nesting situations. Stresses such as this could also activate heat shock proteins such as those that are involved in multi-generational inheritance of bizarre phenotypes in *Drosophila* without a change in DNA sequence. For example, Hsp90 inactivation has been linked to *Ubx* expression and the formation of pollen baskets on the legs of bees. On the other hand, Cini et al. ask how it is that some eusocial species went the other way and lost the sterile caste? Some of these species that showed social reversals evolved into social parasites that still depend on workers, but they exploit workers of closely related eusocial species. It seems more data is needed to determine whether comparing expression levels of conserved genes such as *Ubx* in different castes and species will provide insight into this process.

Comparative studies of social insects and their solitary relatives can be used to look for signatures of social evolution. Sovik et al. analyze the question of whether specific miRNAs may have predisposed bee species to evolve eusociality. One pattern that emerges is that taxonomically restricted genes apparently have the highest rates of adaptive evolution in the honeybee. Similarly, recent expansions of regulatory sequences are restricted to specific ant lineages. A population genomic study combined with a meta-analysis of microarray data in the honeybee suggest that both protein coding and regulatory sequences that are rapidly evolving tend to lie at the periphery of gene networks (Moldostova et al.). One question asked by Helanterä and Uller is whether genes that show biases in expression between morphological castes of ants and bees are under strong purifying selection or whether neutral processes allow genes to be co-opted for specific roles in castes. Similar differences in gene expression have been observed between morphs of plants and animals. More data comparing expression between and within castes is needed to answer these questions.

The final three chapters we will mention take a more mechanistic approach to understanding development and behavior of bees. It has been repeatedly shown that fundamental changes in gene expression during development of either the worker or queen phenotype are mediated by ecdysteroid hormones. An impressive series of experiments by Mello et al. characterize the interactions of ecdysone, juvenile hormone and ecdysone receptor expression, along with downstream gene regulation in the fat body of honeybees. Analysis of interacting miRNAs on differentially transcribed genes during development may provide even more insight into the making of a queen.

Reciprocal hybrids derived from European and Africanized honeybees exhibit both gene expression differences and aggressive behaviors that depend on the direction of the cross. In hybrids with European maternity (but not the reciprocal family), about 8% of genes tested were strongly biased toward expression of the maternal allele in European-maternity hybrids (Gibson et al.). The biased genes are enriched for mitochondrial proteins and genes of metabolic function. Most biased genes are dispersed in the genome but large tracts of them are localized to two quantitative trait loci reported to influence aggressive behavior and alarm pheromone production. The authors speculate that this phenomenon involves partial cytoplasmic incompatibility, nuclear/mitochondrial signaling, heat-shock proteins and short interfering RNA.

The vast majority of social insects are in the order Hymenoptera—the bees, ants, and wasps, which exhibit male haploidy. In most of these species female development is determined by heterozygosity at a single locus but some wasp species rely on a process that signals fertilization of the egg. A common theme however is the involvement of the gene transformer. In honeybees, it appears that duplication of a putative ortholog of *tra*, called *fem*, followed by positive selection resulted in the single-locus, multi-allele complementary sex determiner (*csd*) gene. Biewer et al. present evidence that ancestral duplications of *fem* is restricted to specific bee lineages. They go on to discuss how the gene that sends the initial signal in sex determination could be re-purposed after duplication.

It has been 10 years since the honey bee genome was published. Currently (2016), we have about 50 social insect genomes published with an expected rapid increase in the rate of genome sequencing on the horizon. For example, a proposal to sequence all ant genera has just been put forward by a group of researchers (GAGA, Global Ant Genomics Alliance). Hence, in the near future comparative genomics will greatly increase our knowledge about the processes that shaped the genomes of social insects. For example, comparative studies of bees will be useful for understanding changes associated with the evolution of sociality because there were multiple gains and losses of the eusocial lifestyle in this clade (Kocher and Paxton, 2014). Sequencing of individuals from population studies, coupled with phenotypic data will help identify genes under selection during social evolution, including the genetic architecture of traits of primitively social species. Functional genomics of social insects will be greatly aided by gene editing using CRISPR/CAS methodologies, RNAi and physiological and behavioral assays that are informed by what is learned from metabolomics and transcriptomics will enable social insects to be models for understanding behavioral genetics in general and social evolution in particular.

## Author contributions

All authors listed, have made substantial, direct and intellectual contribution to the work, and approved it for publication.

### Conflict of interest statement

The authors declare that the research was conducted in the absence of any commercial or financial relationships that could be construed as a potential conflict of interest.
